# Production of Human Endothelial Cells Free from Soluble Xenogeneic Antigens for Bioartificial Small Diameter Vascular Graft Endothelization

**DOI:** 10.1155/2015/652474

**Published:** 2015-06-04

**Authors:** Juliana Lott de Carvalho, Alessandra Zonari, Ana Cláudia Chagas de Paula, Thaís Maria da Mata Martins, Dawidson Assis Gomes, Alfredo Miranda Goes

**Affiliations:** Laboratory of Cellular and Molecular Immunology, Department of Biochemistry and Immunology, Institute of Biological Sciences, Universidade Federal de Minas Gerais, 31270-901 Belo Horizonte, MG, Brazil

## Abstract

Arterial bypass graft implantation remains the primary therapy for patients with advanced cardiovascular disease, but most lack adequate saphenous vein or other conduits for bypass procedures and would benefit from a bioartificial conduit. This study aimed to produce human endothelial cells (hECs) in large scale, free from xenogeneic antigens, to develop a small diameter, compatible vessel for potential use as a vascular graft. Human adipose-derived stromal cells (hASCs) were isolated, cultured, and differentiated in the presence of human serum and used for the reendothelization of a decellularized rat aorta. hASC derived ECs (hASC-ECs) expressed VEGFR2, vWf and CD31 endothelial cell markers, the latter in higher levels than hASCs and HUVECs, and were shown to be functional. Decellularization protocol yielded aortas devoid of cell nuclei, with preserved structure, including a preserved basement membrane. When seeded with hASC-ECs, the decellularized aorta was completely reendothelized, and the hASC-ECs maintained their phenotype in this new condition. hASCs can be differentiated into functional hECs without the use of animal supplements and are capable of reendothelizing a decellularized rat aorta while maintaining their phenotype. The preservation of the basement membrane following decellularization supported the complete reendothelization of the scaffold with no cell migration towards other layers. This approach is potentially useful for rapid obtention of compatible, xenogeneic-free conduit.

## 1. Introduction

Approximately one-third of the 500.000 coronary bypass patients operated on in the USA every year [1] lack sufficient or healthy autologous tissue for vascular grafting [2]. Such patients then rely on synthetic grafts, which are a useful alternative to fill such gap, since they present favorable patency properties [2]. Currently, though, these grafts suffer from high thrombogenicity rates for small diameter vessels (<6 mm) [3], leaving a great unmet need for small diameter vascular grafting.

One of the strategies adopted so far to prevent graft failure is to cover graft interface with primary human endothelial cells (hECs), even though this procedure is hindered by low cell yield, limited cell plasticity, and loss of differentiation capacity of hECs following prolonged expansion* in vitro* [4]. In such scenario, the search for alternative sources of hECs is of interest. Human adipose-derived stromal cells (hASCs) have been shown to be easily isolated, expanded, and differentiated into hECs* in vitro*, constituting an excellent option for large-scale production of compatible and functional hECs. However, current cell culture supplements contain xenogeneic proteins, which may be source of infections [5, 6] and generate specific immune responses [7].

Recently, our group has shown that human allogeneic serum (aHS) is a suitable cell culture supplement for hASCs [8]. In addition to successfully maintaining hASCs* in vitro*, it maintains hASCs multipotency and induces higher proliferation rates without promoting cell senescence or loss of differentiation capacity [8–10]. Therefore, the use of aHS may be beneficial for tissue engineering approaches.

In the present study, we aimed to produce functional hECs and use them to promote the reendothelization of rat decellularized aortas. We propose that the association of hECs originated from human adipose-derived stromal cells (hASC-ECs) and a xenogeneic extracellular matrix (ECM) is feasible and may potentially constitute a fast and efficient strategy to develop a compatible vessel free from xenogeneic soluble antigens for potential use as a small-diameter vascular graft.

## 2. Material and Methods

### 2.1. aHS Obtention

The aHS was obtained from the whole blood of healthy donors from all blood group types, as previously described by our group [8]. Briefly, whole blood was collected and allowed to clot spontaneously. Serum was then separated by centrifugation and pooled to produce the aHS. The aHS was inactivated at 56°C for 30 min and aliquoted in sterile tubes, which were kept frozen until use. This procedure was approved by the Ethics Committee in Research from the Federal University of Minas Gerais (number ETIC 21176413.9.0000.5149).

### 2.2. Basal Medium

Basal Medium (BM) consisted of Dulbecco's modified Eagle medium-high glucose (DMEM) (Sigma-Aldrich) supplemented with 5 mM sodium bicarbonate (Cinética Química Ltda), penicillin (100 U/mL), streptomycin (0.1 mg/mL), amphotericin B (0.25 mg/mL; Sigma-Aldrich), gentamicin (60 mg/L; Schering-Plough), and 10% aHS.

### 2.3. Endothelial Medium

Endothelial medium (EM) was composed of DMEM supplemented with 5 mM sodium bicarbonate (Cinética Química Ltda), penicillin (100 U/mL), streptomycin (0.1 mg/mL), amphotericin B (0.25 mg/mL), (Sigma-Aldrich), gentamicin (60 mg/L; Schering-Plough), 1% aHS, 1 mg/mL VEGF (Invitrogen), and 0.1 mg/mL bFGF (Invitrogen).

### 2.4. hASCs Isolation and Culture

Human adipose tissue was harvested from healthy patients, which had abdominal reduction surgery for aesthetic reasons. This study was approved by the Ethics Committee of the Federal University of Minas Gerais (number ETIC 21176413.9.0000.5149). The isolation and culture of hASCs were performed as described, with minor modifications [8, 11]. Briefly, the excess of blood in the raw lipoaspirates was removed with phosphate-buffered saline (PBS) washes and enzymatic digestion of adipose tissue was performed with the incubation of the tissue in a solution of 0.075% type I collagenase I (Life Technologies) in PBS at 37°C for 1 h. Then, the stromal vascular fraction was isolated by centrifugation at 252 g for 10 min, and the pellet was suspended in BM and plated into cell culture flasks (Sarstedt). The flasks were incubated at 37°C in 5% CO_2_ and a humidified atmosphere. After 12–28 h of incubation, plastic adherent cells were isolated with media change and those were termed hASCs. BM was changed every 2-3 days. Subculture was performed every time cell population achieved 80–90% confluency, with incubation of the cells in 0.05% Trypsin-EDTA (Invitrogen) solution for 3–5 min, when cells began to detach from tissue culture flasks. Trypsin solution was then inactivated by adding the same volume of BM supplemented with human serum to flasks, and the resulting cell suspension was replated in new cell culture flasks. The cells were expanded this way until passage 4, when they were used in the assays.

### 2.5. Flow Cytometry

Phenotypic characterization of isolated hASCs was performed as recommended by the Mesenchymal and Tissue Stem Cell Committee of the International Society for Cellular Therapy [12] and performed as already described elsewhere [8]. Briefly, fourth passage undifferentiated hASCs were detached from cell culture surface and counted. About one million cells were then washed with PBS and incubated with the following primary unconjugated mouse monoclonal antibodies: CD29 (Santa Cruz Cat. number sc-9970), CD44 (Santa Cruz Cat. number sc-7297), CD34 (Abcam Cat. number ab8147-500), and CD45 (from BD Biosciences, Cat. number 616256) and with the following conjugated antibodies: HLA-ABC-fluorescein isothiocyanate (FITC) (Abcam, Cat. number ab20313-100) and HLA-DR-FITC (Abcam, Cat. number ab36775-500). Secondary antibodies used were Alexa Fluor 488 goat anti-mouse IgG (Invitrogen, Cat. number A11001). Flow cytometry was performed using a Guava easyCyte 6-2L Flow Cytometer (Millipore). Five thousand events were acquired using the software Incyte (Millipore), analyzed using FlowJo 7.5.6 and compared to isotype controls.

### 2.6. Endothelial Differentiation

Endothelial differentiation was performed with the culture of 7.5 × 10^4^ cells in endothelial medium. Briefly, hASCs were detached from tissue culture flasks, counted, and seeded in BM at a density of 10^3^ cells/cm^2^ in T75 cell culture flasks (Sarstedt). Twenty-four hours later, the BM was removed and EM was used thereafter up to 21 days. EM was changed every 2-3 days.

### 2.7. RT-PCR

The differentiation of hASCs towards endothelial cell fate was assessed according to gene expression of the endothelial markers VEGF receptor type 2 (VEGFR2) and Von Willebrand Factor (vWF). In order to do that, at the end of the induction period, total RNA was extracted using TRIzol and reverse transcripted into cDNA using RevertAidTM H Minus M-MuLV RT. PCR was performed for VEGFR2 (forward primer (FP): 5′GGAATACCCCTTGAGTCC3′, reverse primer (RP): 5′CCTCCAACTGCCAATACC3′), vWF (FP: 5′CGC TCCAGGATGGCTGTG3′, RP: 5′GTACATGGCTTTGCTGGC3′), GAPDH (FP: 5′GGTATCGTGGAAGGACTCATGAC3′, and RP: 5′ATGCCAGTGAGCTTCCCGTTCAGC3′). mRNA was also isolated from human umbilical vein endothelial cells (HUVECs) as positive control. PCR cycles were as follows: 94°C for 2 min, 94°C for 30 s, 56°C (for vWF and VEGFR2) and 60°C (for GAPDH) for 45 s and 72°C for 45 s (30 cycles), and 72°C for 10 min. The RT-PCR products were analyzed through 1% agarose gel electrophoresis and visualized with ethidium bromide.

### 2.8. Immunofluorescence

At the end of the differentiation period, cells were stained for endothelial markers. Immunofluorescence was performed following cell fixation with 4% paraformaldehyde. Afterwards, cells were washed, permeabilized, and incubated with primary antibodies overnight at 4°C. Cells were then washed and incubated with secondary antibodies for 1 h at room temperature. Finally, cell nuclei were stained with DAPI and slides were mounted using Hydromount. Primary antibodies used were mouse anti-vWF (Abcam, Cat. number ab129948) and rabbit anti-VEGFR (Abcam, Cat. number 2349-500). Secondary antibodies used were Alexa Fluor goat anti-mouse 488 (Molecular Probes, Cat. number A21428), and Alexa Fluor goat anti-rabbit 555 (Molecular Probes, Cat. number A11001). HUVECs were stained and considered positive control.

The same antibodies were also used for tissue immunostaining of the recellularized aortas. Tissue sections were washed in water and protocol was similar to described for cells.

### 2.9. Tube Formation Assay

The capacity of hASC-ECs to form tubes in matrigel was assessed as previously described as an* in vitro* assay to assess endothelial cell function [13]. For this assay, 4 × 10^4^ cells were seeded on each well of a 24-well plate coated with 225 *µ*L of previously solidified Matrigel (BD Biosciences) and incubated overnight at 37°C in 5% CO_2_ incubator. Cells were then incubated in 3 *µ*g/mL Calcein-AM (Invitrogen) diluted in PBS for 30 min at 37°C in 5% CO_2_ incubator, protected from light. Finally, tube formation was observed, quantified and tube area was measured both under light microscopy and under fluorescence microscope, using the ImageJ 1.47 software.

### 2.10. Aorta Decellularization and Animal Information

Lewis LEW-Tg (EGFP) F455.5/Rrrc rats 6–8 weeks old, which express enhanced fluorescence green fluorescent protein (EGFP), were obtained from the Rat Resource and Research Center, Missouri, USA. The animals were housed in a climate-controlled environment under a 12 h light/dark cycle with free access to rat chow and water. All experimental protocols were performed in accordance with the guidelines for the humane use of laboratory animals established at our Institution. This study was approved by the Committee of Ethics in Research at the Federal University of Minas Gerais (Protocol number 140/2011).

Aorta decellularization was performed according to the protocol published by [14], with minor modifications, as described by [15]. Briefly, the isolated aorta was perfused with 1% SDS and 1% Triton X-100 solutions and sterilized by perfusion of PBS supplemented with 100 U/mL penicillin-G, 100 U/mL streptomycin, and amphotericin B. After, Aorta was isolated from the heart and cannulated in its other end to mount a closed perfusion system. Following decellularization, approximately 50 mg of tissue was processed using DNAzol reagent (Life Technologies) for genomic DNA isolation, following manufacturer's instructions. Isolated DNA was quantified using Nanodrop ND-1000 spectrophotometer (Life Technologies, USA).

### 2.11. Aorta Reendothelization

hASC-EC were evaluated according to their potential of reendothelizing a decellularized aorta in the presence of EM with human serum. In order to do so, endothelial cells were harvested and counted. 2.5 × 10^6^ cells were then perfused into the decellularized matrix with an initial static period overnight, followed by 7 days of continuous perfusion with EM.

### 2.12. Decellularized Aorta Processing for Histology and Immunofluorescence

Following fixation in 4% paraformaldehyde, aortas were embedded in paraffin or OCT (Optimal Cutting Temperature). 5 *μ*m tissue slices were cut for histology and immunostaining. Hematoxylin and Eosin staining was performed. ECM proteins laminin and collagen I were stained following tissue slice production, following the same steps of immunofluorescence cell staining. Primary antibodies used were mouse anti-rat collagen I and rabbit anti-rat laminin (both from Abcam, UK). Secondary antibodies were the same used for cell staining.

## 3. Results

### 3.1. hASCs Characterization

**Figure 1:**
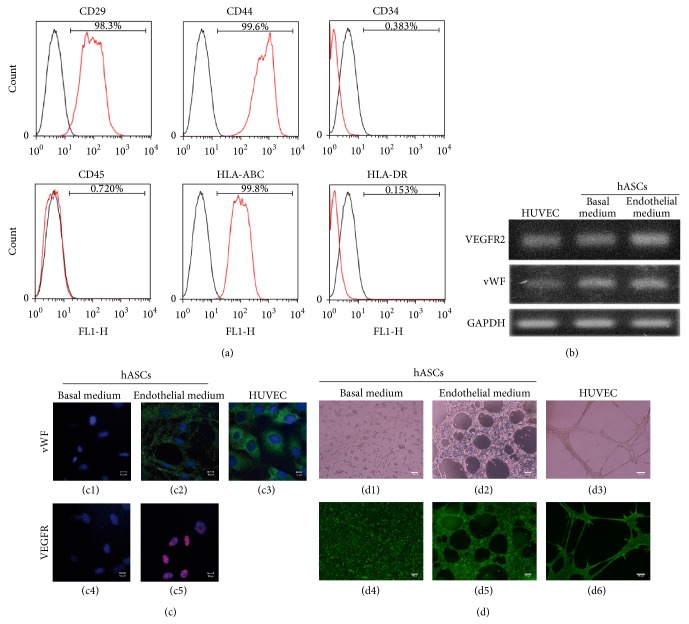
hASCs characterization and endothelial differentiation. (a) Flow cytometry analysis of hASCs expression of mesenchymal stem cell markers. Black line indicates isotype-matched monoclonal antibody controls and red line indicates positive stained cells. (b) RT-PCR analysis of VEGFR2 and vWF mRNA expression during endothelial differentiation of hASCs. The expression of those markers was also assessed at protein level by immunohistochemistry (c). Green fluorescence corresponds to vWF protein presence (c1, c2, and c3), red corresponds to VEGFR protein expression (c4 and c5), and blue corresponds to cell nuclei (c1–c5). (d) Functional analysis of endothelial cell differentiation, as assessed by tube formation assay. In (d1) to (d3), inverted microscopy was used to document tube formation. In (d4) to (d6), cells were stained with calcein-AM and observed under fluorescence microscope, in order to show cell viability in addition to tube formation. *n* = 3 for each experiment.

Isolated hASCs cultured in BM were positive for CD29 (98.3%) and CD44 (99.6%) and lacked the expression of hematopoietic stem cell-associated markers CD34 (0.38%) and CD45 (0.72%). In addition, hASCs expressed HLA-ABC (99.8%) and lacked HLA-DR (0.15%) (Figure 1(a)).

Functionally, isolated hASCs proved their multilineage differentiation potential and were capable of generating osteoblasts, chondrocytes, and adipocytes following differentiation (data not shown). Those were capable of mineralizing the ECM, depositing proteoglycan in ECM, and storing lipid droplets in their cytoplasm, respectively. Therefore, isolated hASCs attended to the criteria of mesenchymal stromal cell (MSC) characterization both phenotypically and functionally.

### 3.2. hASCs Endothelial Differentiation in Induction Medium Containing Human Serum

Endothelial differentiation of hASCs in the presence of aHS was confirmed by gene and protein expression of endothelial markers; therefore, they were regarded as hASC-ECs. First, expressions of VEGFR2 and vWF were assessed by PCR. As expected, hASC-ECs were capable of expressing both markers related to the endothelial lineage. Undifferentiated hASCs also expressed such genes in discrete smaller levels, similar to HUVECs, which were used as positive control (Figure 1(b)). In a quantitative approach, the expression of CD31 was also assessed (Figure  S.1 in Supplementary Material available online at http://dx.doi.org/10.1155/2015/652474) and indicated that hASC-ECs had approximately 11 times higher CD31 expression compared to both hASCs and HUVECs, which had statistically similar expression of this marker, even though the latter had a slightly higher CD31 expression level then hASCs.

The expression of both VEGFR and vWF was assessed at protein level by immunofluorescence. Our results show once again the consistent differentiation of hASC-ECs into the endothelial phenotype, as both hASC-ECs and HUVECs exhibited VEGFR and vWF proteins. hASCs presented no expression of those proteins, evidentiating the difference between undifferentiated hASCs and their hASC-ECs derivatives (Figure 1(c)).

### 3.3. Tube Formation Assay

One of the functional assays to assess endothelial differentiation consists on investigating the capacity of hECs of forming tubes when cultured in Matrigel in the presence of stimulus. Since the undifferentiated hASCs express VEGFR and vWF in discrete levels, this assay is necessary to differentiate hASCs from functional hECs. To do so, first, we cultured HUVECs in such condition and showed their capacity to form tubes. Following, hASCs and hASC-ECs were also used in the experiment and only differentiated hASCs (hASC-ECs) and HUVECs were capable of forming tubes once stimulated in Matrigel (Figure 1(d)). Even though hASCs express some hECs markers, those are not present at the protein level and hASCs are not able to form tubes when seeded on Matrigel. Cell viability was assessed with Calcein-AM. The qualitative observation was confirmed by quantitative analysis of the relative numbers of tube formation and the median size of those tubes. Once again, HUVECs showed high capacity of forming tubes, presenting an average of 8 per field, with average size of 22431 *μ*m^2^. HUVECs were closely followed by hASC-ECs, which were also able to generate tubes under the tested conditions, with 14 tubes per field (average size: 8599 *μ*m^2^). Tube formation by hASCs was not observed and considered virtually absent.

### 3.4. Aorta Reendothelization

**Figure 2:**
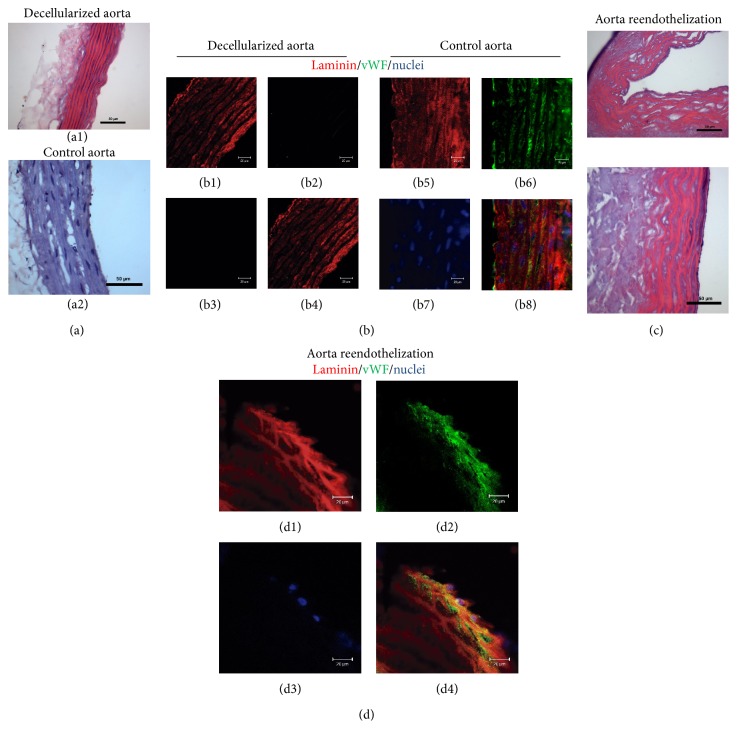
Decellularization of rat aortas and recellularization with hASC-ECs. (a) Perfusion-decellularized rat aortas (a1) presented intact ultrastructure but were devoid of cell nuclei, in contrast to nondecellularized control rat aortas (a2). (b) Decellularization process (b1 to b4) did not compromise laminin content, as compared to nondecellularized control aorta (b5 to b8). (b1) and (b5) correspond to laminin staining (red), (b2) and (b6) correspond to vWF staining (green), (b3) and (b7) show cell nuclei content (blue), and (b4) and (b8) are image overlays. (c) Recellularized rat aortas present continuous endothelium in their inner surface (c2), absent in decellularized controls (c1). Recellularization with hASC-ECs rebuilt the endothelium of decellularized aorta, which maintained laminin content (red, d1) and regained vWF (green, d2) and nuclei (d3) in their endothelium. Image overlay (d4). *n* = 3 for each experiment.

Finally, the reendothelization of a decellularized aorta was performed in order to assess the applicability of using hASC-ECs to promote construct viability* in vivo*. First, aorta decellularization was verified by histology and DNA isolation. As expected, decellularized aorta was shown to be deployed of cell nuclei and DNA. Additionally, decellularized aortas presented laminin preserved along their layers, including the basement membrane (Figures 2(a) and 2(b)).

After a period of static seeding, hASC-ECs were reperfused and cultured for 7 days in the closed system in order to attach and proliferate in the matrix. Following such period, cells formed continuous sheets of viable cells, as shown by histology and immunofluorescence (Figures 2(c) and 2(d)). Even after 7 days of culture in a perfusion biorreactor, hASC-ECs still expressed VEGFR and vWF and strongly adhered to the basement membrane of the decellularized aorta (Figure 2(d)), evidentiating the stability of the construct in a perfusion system for 7 days.

## 4. Discussion

The search for vascular substitutes has proven to be difficult, specially when considering small diameter grafts. Major problems concerning vascular engineering comprise thrombogenesis, calcification, and patency maintenance. An effective strategy to prevent such events to occur is to protect materials from circulation by covering them with hECs. Therefore, disregarding the vascularization strategy used to substitute occluded or diseased vessels, hECs are always a key component. A continuous endothelium on the flow surface maintains graft patency and prevents platelet deposition, thrombosis, and neointimal hyperplasia [16]. Also, it excludes the need of fixation steps and prevents the presence of cell debris and the attraction of calcium by glutaraldehyde, both related to the process of calcification [17]. Beyond that, hECs are in direct contact to several lymphoid cell types and may initiate rejection in an allograft context, functioning as antigen presenting cells and directly activating lymphocytes [18]. Therefore, they must not be different from host nor present xenogeneic antigens.

Endothelization of vascular grafts is observed* in vivo* within weeks to months postgrafing. Monoclonal antibodies, recombinant proteins, and aptamers have been used to accelerate graft endothelization [19]. We propose that endothelization* in vitro* is faster and more efficient, since it has been shown that despite being reendothelized* in vivo* vascular grafts lacking a continuous endothelium still show thrombosis and calcification. In this scenario, obtaining high numbers of autologous hECs is imperative. hECs can be easily isolated from patients as circulating progenitors [20, 21] or from vessel wall biopsies [22, 23]. However, those approaches are hindered by low cell yield, limited plasticity, and differentiation capacity following prolongated expansion* in vitro* [24]. Here, we show that adipose tissue constitutes a promising cell source for hECs generation, as it does not suffer from the aforementioned limitations. In addition, adipose tissue may prove to be a more reliable source of autologous stem cells than blood or bone marrow, where the presence of diabetes and other comorbidities may significantly decrease the availability of stem cells for clinical use.

hASCs are easily isolated from adipose tissue and have a clear potential for clinical applications. Indeed, several clinical trials using these cells have been performed aiming for the treatment of various injuries and diseases (clinicaltrials.gov). hASCs may be obtained from the patient in times of health, in order to be used when needed, or from healthy donors. Therefore, the isolation of hASCs and further differentiation towards endothelial lineage constitute faster and more reliable approach for tissue engineered vascular graft (TEVG).

hASCs endothelial differentiation* in vitro* is consistent and reproducible [25–27]. Most differentiation protocols are based on induction with VEGF and bFGF [25, 27]. Even though the mechanism for hECs induction is not clear, it is known that VEGF induces hECs differentiation in a Rho-GTPase-dependent manner. Physical stimuli (shear stress) also promote hASCs endothelial differentiation [26, 28]. In the present work, we associate both stimuli for hASCs differentiation. Our results indicate that generation of hASC-ECs in the presence of aHS was succesful. hASC-ECs presented CD31, VEGFR2 (also known as KDR), and vWF. Among those markers, CD31 is the most specific, since KDR may be expressed in progenitors and pluripotent stem cells [29, 30], and vWF may be released by platelets [31]. Therefore, CD31 expression was assessed quantitatively, by qPCR (Figure  S.1), and revealed that hASC-ECs had the higher expression of this surface marker among samples tested, which also included hASC and HUVECs. This data can indicate that the HUVEC cell line maintained in our lab may have lost the expression of such marker or that maybe our culture conditions did not promote the maintenance of CD31 expression. Nevertheless, other markers were assessed and shown to be present at HUVECs, in addition to hASCs and hASC-ECs, both at the mRNA and protein levels. Those markers were VEGFR2 and vWF. Even though those markers can be related to other events, it is known that the former plays a major role in the* in vivo* angiogenesis and contributes with matrix-metalloproteinase to the formation of capillary-like structures seen* in vitro* [29]. The latter mediates the adhesion of blood platelets at sites of vascular injury, stabilizes clotting factor VIII, and contributes to hECs anchoring to ECM [30]. Even though they are also expressed in discrete amounts at mRNA level in hASCs, those markers are not present at the protein level. Cell populations under investigation in the present work were also assessed in terms of functionality, in order to further strengthen our claim of having generated hASC-ECs in nonxenogeneic conditions. As expected, hASCs are not functional hECs and were unable to generate tubes when seeded on Matrigel. In contrast, hASC-ECs were able to generate tubes under the tested conditions, confirming their endothelial phenotype, as well as HUVECs. Even though hASC-ECs formed tubes in higher amounts and smaller diameters compared to HUVECs, those differences emphasize the higher proliferative potential of hASC-ECs [31], which is a positive feature, considering the proposed objectives of the present work. This assay also confirmed that the HUVEC cell line maintained in our lab may have lost CD31 expression but is still functional and is representative of the endothelial phenotype.

The seeding of hASC-ECs in biomaterials and decellularized matrices has been performed with exciting results [19, 31]. It has been described that hEC coated TEVG is feasible and safe, but the developed strategy used autologous primary cultures of hECs and took several weeks to achieve complete graft endothelization [32]. With our approach, this time can be shortened, due to high proliferation rates of hASCs cultured with aHS.

The production of the aforementioned TEVG requires the investigation of ways to exclude soluble animal antigens from cell culture, before performing hASCs differentiation. Use of animal origin reagents is controversial due to the possibility of zoonosis disease transmission. The risk of prion infection can be avoided, but the use of an average of 20% FBS in cell culture, regardless of the type, results in MSC carrying between 7 and 30 mg of bovine serum proteins, resulting in specific immune reaction in humans [33].

Animal-free alternatives have emerged for immortalized cell cultures, but few products exist for primary adult human cells, and they typically provide suboptimal results. Previously, our group has described that allogeneic human serum is a suitable supplement for proliferation and differentiation of hASCs [8]. In this work, the results indicate that hASCs not only maintained their classic phenotype in such condition (e.g., cell markers and multipotent differentiation capacity), but also were able to differentiate into functional hECs, as shown by the tube formation assay. Finally, functional hECs were able to generate a continuous cell sheet in the preserved basement membrane of a rat aorta, in the presence of shear stress. Here, we generated functional hECs free from animal soluble antigens and showed that they are functional and able to be used for TEVG approaches, in a potentially faster and safer manner.

## Supplementary Material

Supplemental File 1 presents the difference in expression of CD31 among hACSs, hASC-ECs and HUVECs, as assessed by qPCR.
