# Supplementary material for: Production of Human Endothelial Cells Free from Soluble Xenogeneic Antigens for Bioartificial Small Diameter Vascular Graft Endothelization
Source: Biomed Res Int. 2015 Jun 4;2015:652474. doi: 10.1155/2015/652474 (PMC4471259; doi:10.1155/2015/652474)
Supplement: Supplementary file 1 — Supplemental File 1 presents the difference in expression of CD31 among hACSs, hASC-ECs and HUVECs, as assessed by qPCR. [file 652474.f1.pdf]

SUPPLEMENTAL MATERIAL

SUPPLEMENTAL MATERIALS AND METHODS

qPCR

Total RNA was extracted from hASCs, hASC-ECs and HUVECs, using Trizol, as described by the manufacturer. Total RNA samples were then treated with DNase (Promega), according to usage information. A NanoDrop ND-1000 Microspectrophotometer was used to measure the total RNA amount (ng/µL). 1 ug of each sample was treated with the RevertAidTM H Minus M-MuLV RT (Fermentas) to generate cDNA using an oligo(dT) adapter primer. Next, qPCR amplification was performed for the CD31 and GAPDH genes.

qPCR was performed using the 7900 Real-Time PCR System (Applied Biosystems) using SYBRGreen Master mix (Appllied Biosystems). The following primers were used: GAPDH - FP: ACATCGCTCAGACACCATG, RP: TGTAGTTGAGGTCAATGAAGGG; CD31 - FP: ACGGAAGTTCAAGTGTCCTCAG, RP: GCTTTCCACGGCATCAGGGA. The relative level of gene expression was determined by the comparative cycle threshold (Ct) method with each sample normalized to GAPDH and expressed as a relative change. Each sample was run in triplicate. Results were averaged and expressed as relative amounts. Comparisons were made between samples processed at the same time.

Statistical analysis

Statistical analises were performed using Graph Pad Prism 5.0. Values were presented as mean ± standard deviation. Groups were compared by analysis of variance and Bonferroni's post-tests. Differences were considered significant at $p < 0.05$.

SUPPLEMENTAL FIGURE

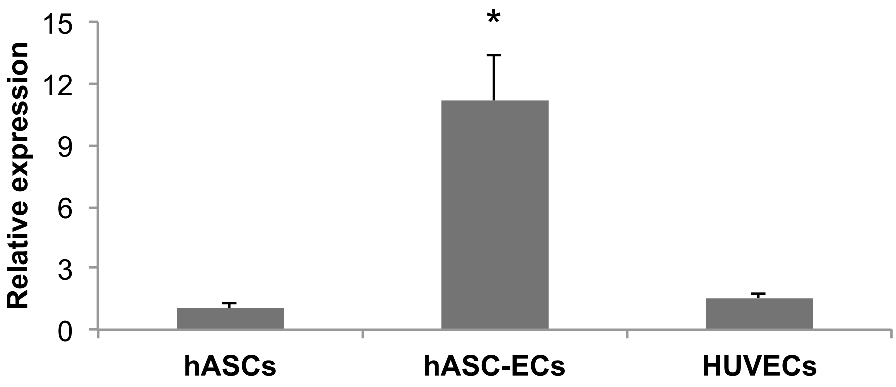

Figure S.1: Relative expression of CD31. CD31 expression, normalized to GAPDH, was assessed by qPCR in undifferentiated hASCs, differentiated hASC-ECs and HUVECs. *p<0,05 compared to control (hASCs).
